# Sickness in Europe

**Published:** 1865-06

**Authors:** 


### SICKNESS IN EUROPE.

Europe seems to be getting into a panic in view of the fact that in Eastern Europe there is at present prevailing an unusual amount of sickness, with a tendency to spread westwardly. It is somewhat remarkable that most of the great epidemics recorded in history, have moved from east to west. The great plagues of the fifth century, B. C., and of the second, fourteenth and seventeenth centuries, of the Christian era, and the cholera of the fourth decade of the present century, all attest this fact.

The accounts of the disease now prevailing in Russia are at present somewhat confused, so far as they have fallen under our notice. There seems to be an effort to conceal the fact as much as possible, that there is any special sickness prevalent. A correspondent of the London *Times*, writing from Berlin, says that there appears to be three several maladies prevailing at the same time in St. Petersburg. 1. In October last, spinal meningitis broke out. This disease seems to answer the description of the cerebro-spinal meningitis, or "spotted fever," that has been so prevalent and so fatal in some portions of this country. The mortality of this disease is stated at from twenty to fifty per cent. 2. Typhus fever followed in November. This occurred sporadically at first, and gradually developed into a malignant species of recurrent fever. The fever lasts a week at a time, the several attacks being separated by intervals as long. During these intervals, the health is apparently so good that people have been dismissed from the hospital, who died soon after. A special committee has been formed under Governor-General Suwaroff to look after those apparently cured. On a second or third attack, there is a general collapse, decomposition of blood, and paralysis. Quinine and stimulants have no effect. The deaths, at first but twenty, have risen to forty per cent. The spleen and liver are much affected. In many cases epidemical inflammation of the spleen, with *pustula maligna*, has been observed.

It would seem to be in reference to this disease that the British ambassador in St. Petersburg says:—"the fever is contagious, and is called in French, *fièvre à rechute*, in German, *das recurrirende Fieber*, and 'the relapsing or famine fever' in English. It is also styled 'remittent fever,' or 'typhus recurrens,' or 'bilious typhoid fever,' or 'synocha,' or 'miliary fever,' or 'typhinia.' It was unknown in Russia until eight months ago, when Professor Botkin called attention to it as a fever he had never seen, though foreign physicians had described it."

3. But more recently another disease, it is represented, has appeared, and it is this which seems to be creating such a panic in Europe. The malady is stated to have broken out originally on the other side of the Ural Mountains, whence it slowly threaded its way toward St. Petersburg, increasing as it went, and culminating at length among the destitute classes of an unhealthy and densely populated capital. In its steady advance toward the west it has now reached the Prussian frontier, and, in a milder form, already shown itself in the towns of Konigsberg, Dantzig, and Gumbensen. In the Waldai hills, to the south-west of St. Petersburg, whole villages are said to have been depopulated.

A Paris correspondent of a New York paper says:—"A description of the symptoms and course of the disease which reached here, at first seemed to me to be nearly the same as those in violent cases of yellow fever,—sudden pain in the nape of the neck, in the hand, or foot, or, indeed, any part of the body, followed by violent chill and fever; a second chill coming on the following day, with increased violence. This paroxysm is frequently fatal, a third invariably so. Another peculiarity, which is common in yellow fever, marks the Siberian plague, as it has been called. Patients who appear to be entirely recovered, at the end of five or six days are subject to a fatal relapse if they leave their beds."

The *Medical Gazette* of St. Petersburg, is quoted as saying —"It was during last autumn that the disease broke out simultaneously in the northeast and southwest of the Russian empire. M. Gworling, the government doctor at Perm, announced that he had seen forty cases of this disease, which commences with cold and shivering, followed by great heat, without perspiration, accompanied by colic, headache, delirium, general debility, and sometimes with a flow of blood from the nose. The disease lasts from five to six days, after which the invalid recovers his appetite, falls into a sound sleep, and the fever quits him. The fever returns after a short interval, but again ceases, and the doctor has observed this to have happened three or four times. Quinine taken in large doses cures the fever, and removes delirium, but does not produce decisive effects, nor is it sufficient to arrest the progress of the disease."

One correspondent speaks of extreme dilation of the pupils, a strong disposition to vomit, which can not be satisfied, a swelling of the abdomen, pestilential carbuncles, and dark color of the skin, as its unmistabable symptoms.

A Florence journal publishes a communication from Dr. Galligo, which gives the most connected medical account of the epidemic that we have yet seen, though still very incoherent and unsatisfactory. Dr. Galligo says:—"We have received from Dr. Tilleur, physician to the Grand Duchess Maria of Russia, who has just arrived from St. Petersburg, some important details respecting the disease now raging in that capital. This malady appears to be neither a fever of an intermittent or continuous nature, nor yet a simple typhoid fever; but it certainly is very virulent and dangerous. According to the opinions of the Russian physicians, it is the same fever that was observed for the first time in Scotland, in the year 1819, and denominated in that country the intermittent fever, from the length of the intermissions and the prolonged attacks. This fever is ushered in by cold shivering, alternating with remarkable heat (from 40 deg. to 41 deg. centigrade, or 106 deg. Fahrenheit,) the pulse beating 130. Great prostration and disorder are observable in the nervous actions, although the state of the mental faculties remains unaltered; frequent pains are felt in the head and limbs, great pain is also felt in the left hypochondria region, and an examination by palpation and percussion proves the spleen to have immensely increased in volume. The skin is yellow in color, owing to the liver being likewise affected by the malady. The initiatory attack of the fever lasts from seven to eight days, and terminates with a very copious perspiration. After the first paroxysm, an interval occurs of seven or eight days, during which the patient appears almost as well as ever, but at the expiration of that period a second attack manifests itself like the first, but accompanied with still greater prostration. This continues also about seven days, terminating, like the other, with profuse perspiration. Sometimes a third paroxysm declares itself after a further interval of seven days, one of the symptoms being a burning thirst and complete aneurism,(?) and the patient sinks into the most profound state of prostration. The rate of mortality is eight* per cent., and the victims of this malady die during the second attack, usually from a kind of general paralysis, or through serious derangement of the nervous organs, with real decomposition of the blood, and an enormous increase in the spleen. The liver also becomes greatly enlarged, but the intestines, on the other hand, are either found healthy or else hardly congested. Every thing has hitherto failed to shorten the duration of the febrile attacks. Salts of quinine, given in large or small doses, have been quite ineffectual to overcome the attacks characteristic of this malady. In the second paroxysm, in which there is increased prostration of the forces, the most powerful stimulants have been administered—such as moss wine, alcohol, ether, camphor, etc.; but they produce little or no effect. The chief cause of this disease is supposed to be the arrival in St. Petersburg of an immense number of workmen from the neighboring provinces, and even from the most distant towns. It is said there are just now in the capital, 43,000 workmen more than the usual number. The consequence of this is, that they can not find work, and are obliged to live in unhealthy localities, and to live upon the black bread, which contains this year much more horned rye than in previous years. It has been discovered by chemical analysis that this bread contains one per cent. of horned rye in the flour with which it is made. Thus every workingman living on the same, may be calculated to eat 100 grains of horned rye per diem. Besides this, the oxen, cows, and other animals, being no longer slaughtered in St. Petersburg, but in Moscow, whence the meat is despatched ready prepared, the heads, hoofs, feet, and intestines of these animals, which previously formed one of the staple articles of sustenance of the poorer classes, on account of their cheapness, are no longer to be had at St. Petersburg, and the poor are now compelled to live almost exclusively upon the above mentioned bread, which contains injurious substances,

* This must be an error. Another account places the mortality at *seventy per cent.*

partly contributing to produce the disease in qüestion. The malady is exclusively confined to the poorer classes.

"The authorities of St. Petersburg have ceased publishing official returns of the number of cases and mortality, but from the large sums voted by the metropolitan authorities or supplied by government, some idea may be formed of the extent and virulence of the malady. Besides 200,000 roubles (about $160,000) contributed by the treasury, 400 additional beds have been placed at the disposal of the town, and large subscriptions made by the princes and aristocracy. The town, too, has opened a new hospital, at the cost of 60,000 roubles, (about $48,000,) considerably augmenting at the same time the funds of the various charities, and aiding the convents in the care and reception of the sick."

We have given above, the best account we can collate from the materials at our hands, of this "Siberian plague," as it is called, and our readers will perceive in it evidences of the state of panic that exists in regard to it. Later accounts represent the disease as disappearing.

A great many medical men and students have, it is said, gone from Western Europe to St. Petersburg, to study into the nature and habits of the disease, and it is represented that forty or fifty of them have fallen victims to their zeal. Among its victims is mentioned Dr. Erichson, surgeon to the Emperor Nicholas, aged seventy-five.

The western nations of Europe are enforcing quarantine regulations against vessels from Russian ports, and we doubt not that proper precautions will be taken against the importation of the disease into this country. If this, or any other epidemic should be introduced into New York, in its present sanitary condition, and with such inadequate sanitary regulations as it is now subject to, the effects on the country would be deplorable. Then would be *felt* the necessity for such a Health Law, as for several years past has been before the Legislature of that State, and which has by some means been defeated each year.

An attempt is made in some quarters to inculcate the idea that some terrible epidemic will follow our rebellion—that plagues constitute a part of the natural history of rebellions. The way to meet any supposed danger from epidemic diseases is to enforce personal and municipal cleanliness, and to have plenty of air and light in our habitations.—*Med. and Surg. Reporter.*

